# Video- assisted thoracoscopic lung resection with or without enhanced recovery after surgery: a single institution, prospective randomized controlled study

**DOI:** 10.3389/fonc.2024.1474438

**Published:** 2024-11-08

**Authors:** Yi Ding, Leiming Zhou, Lei Shan, Weiquan Zhang, Peichao Li, Bo Cong, Zhongxian Tian, Yunpeng Zhao, Xiaogang Zhao

**Affiliations:** ^1^ Department of Thoracic Surgery, The Second Hospital of Shandong University, Jinan, China; ^2^ Department of Thoracic Surgery, People’s Hospital of Laoling, Laoling of Dezhou, China

**Keywords:** enhanced recovery after surgery, lung resection, thoracic surgery, postoperative pulmonary complications, day surgery

## Abstract

**Purpose:**

This study was conducted to evaluate the postoperative short-term outcomes of patients undergoing video-assisted thoracoscopic surgery (VATS) for lung resection with the enhanced recovery after surgery (ERAS) protocol.

**Methods:**

A single-institution, prospective randomized controlled study was conducted. The primary outcome measures were postoperative pulmonary complications (PPCs) and postoperative short-term effects.

**Results:**

Among the 611 patients, 305 were assigned to the ERAS group, and 306 were assigned to the routine group. The ERAS group achieved earlier oral feeding, earlier mobilization, a shorter duration of drainage (2.0 vs. 5.0 days, P<0.001), and a shorter hospital stay (3.0 vs. 7.0 days, P<0.001). The biological impacts were confirmed to be significantly better for the ERAS group. Furthermore, the ERAS group also had a lower incidence of PPCs (11.5% vs. 22.9%, P<0.001) than did the routine group. Multivariate logistic regression analysis revealed the following predictors of drainage tube removal on the 1st day after surgery without pneumonia during hospitalization: comorbidity (P=0.029), surgical procedure (P=0.001), and operation time (P=0.039).

**Conclusions:**

Implementation of the ERAS protocol led to a decreased incidence of PPCs, suggesting that the ERAS protocol has a better biological impact on patients undergoing VATS for lung resection. Multigradient individual ERAS protocols are recommended at different institutions according to the individual conditions of patients.

**Clinical Trial Registration:**

https://register.clinicaltrials.gov/prs/app/action/SelectProtocol?sid=S0009ZT9&selectaction=Edit&uid=U0002ZGN&ts=3&cx=ks7hrg, identifier NCT04451473.

## Introduction

Enhanced recovery after surgery (ERAS) was first reported in the late 1990s (1). ERAS strategies involve all aspects of perioperative care, aiming to improve patient prognosis, reduce complications, shorten hospital stays, and lower costs (2–6). The concept of ERAS has been applied in open or minimally invasive surgeries, including colorectal surgery (7), gynecological surgery (8), liver surgery (9), breast surgery (10), urological surgery (11), and spinal surgery (12).

Thoracic surgery is considered an invasive and traumatic procedure for patients (13). Minimally invasive surgeries, including video-assisted thoracoscopic surgery (VATS), have been widely used for surgical treatment (14, 15). Thoracic surgery has undergone two major advancements: one is the switch from open surgery to minimally invasive surgery, and the other is the use of structured clinical pathways based on ERAS guidelines (16, 17). Studies have also focused on ERAS strategies for thoracic surgery in recent years (18), especially in VATS for lung resection (19, 20), indicating that ERAS can efficiently minimize surgical trauma, improve the quality of postoperative recovery, and decrease financial burdens. However, the outcome data of ERAS programs applied for lung resection are limited, and more diverse regions of study are needed to determine the safety and effectiveness of ERAS strategies. The aim of this study was to determine the impact of the ERAS pathway on the short-term outcomes of patients who underwent lung resection.

## Patients and methods

### Patient eligibility

The inclusion criteria for patients were as follows (1): had lung lesions suitable for VATS, including benign and malignant lesions diagnosed by CT (enhanced or nonenhanced) or pathological results (2); were aged between 18 and 85 years (3); had a Karnofsky score ≥80 along with cardiopulmonary function, liver function, and renal function indicating the ability to tolerate minimally invasive surgery (4); had normal cognitive function and was able to cooperate with the rehabilitation training (21, 22); and (5) agreed on the protocol of the clinical trial and signed the consent form.

The exclusion criteria for patients were as follows (1): refused randomization (2); was unable to cooperate with the rehabilitation training or tolerate minimally invasive surgery due to cognitive or physical dysfunctions; and (3) participated in other clinical trials or had received treatment with anticancer drugs in other clinical trials.

### Pretreatment workup

The study was approved by the Ethics Committee of the Second Hospital of Shandong University (SDDXDEYY-KYB-2020053). The study’s registration information is as follows: Clinical Trials. Gov ID: NCT04451473 (30/06/2020). Informed consent was obtained from all the patients. Our work was fully compliant with the CONSORT criteria, and this study is reported in line with the CONSORT criteria (23). We confirmed that all methods were performed in accordance with the relevant guidelines and regulations.

### Randomization and allocation

This study was designed as a single-center, randomized, unblinded control trial. Random assignment was performed using the envelope method by a statistician at the Evidence-based Medicine Center of the Second Hospital of Shandong University. Once an informed consent form was signed, the patient was assigned to one group by opening the sequentially numbered envelope (24).

### Study interventions

#### Control group (routine group)

The patients were randomly assigned to the control group (routine group) and received standard VATS; however, the perioperative management was mainly traditional without systemic ERAS protocol guidance. The traditional management methods were as follows: 1. the patient received no systemic physical pulmonary training before surgery; 2. the patient received sedatives to reduce anxiety preoperatively; 3. the patient fasted from solids for at least 8 hours and from liquids for at least 6 hours; 4. a transurethral catheter was routinely placed and then removed on the second day after surgery; 5. the patient remained completely supine for 6 hours after surgery; 6. the patient fasted from solids and liquids for 6 hours after surgery; 7. the patient achieved ambulation ≥24 hours after surgery; 8. two chest tubes were used when the upper lobe was moved; 9. the chest tube was removed when the highest volume did not exceed 100 ml/24 h; and 10. opioids were the most common drugs in the analgesic regime.

#### Intervention group (ERAS group)

The ERAS protocol was performed mainly according to two guidelines (16, 17), and physical pulmonary training in the general ward was recommended according to the protocol (25). The core items of ERAS application at our institution are shown as following:

1. Dedicated preoperative counseling and education should be given in the preoperative phase. Nutritional status screening and improvement, smoking cessation (2-4 weeks), alcohol dependency management, and even pulmonary prehabilitation (according to the patient’s cognitive ability and compliance) should also be accomplished in this phase.

2. Airway management during the preoperative examination after admission: climbing stairs or power-based cycling, which can enhance cardiopulmonary function, has often been used in the past few years; however, physical pulmonary training in the general ward is strongly recommended (25), especially during the COVID-19 pandemic, which has been ongoing since 2020. Education and training are better guided by a physiotherapist or by a charge nurse.

3. Aspirin withdrawal is not recommended for patients in the ERAS group unless VATS is complicated and accompanied by a high risk of bleeding. Low molecular weight heparin bridging treatment is used during aspirin withdrawal.

4. Sedatives for reducing anxiety preoperatively are prohibited.

5. Clear fluids are allowed up until 2-4 h before the induction of anesthesia, and oral carbohydrate loading can be used routinely.

6. A combination of regional and general anesthetic techniques should be used, and general anesthesia combined with nonintubated spontaneous breathing can be an alternative to double-lumen intubation if the anesthesiologist has mastered the technique.

7. Uniport VATS was routine for patients in the ERAS group. Wedge resection or segmentectomy should be meticulously planned through the scientific reading of thin-slice CT or 3D simulation before surgery. High-quality minimally invasive surgery is the foundation of ERAS protocol implementation.

8. A transurethral catheter is not routinely placed for the sole purpose of monitoring urine output without thoracic epidural anesthesia. Transurethral catheters can be used in one of the following conditions (1): the estimated operation time is more than 150 minutes or even 180 minutes (2); high risk of bleeding; and (3) critical patients with organ dysfunction. If the operation takes a longer time than expected without a urinary catheter, placing a disposable catheter is reasonable. The urinary catheter should be removed immediately after the operation if the patients’ respiratory and circulatory status is stable without prediction advanced life support.

9. The head of bed (by ≥30°) was immediately raised when the patient went back to the general ward. The patient could sit up straight with the help of the charge doctor or charge nurse 2 hours after returning to the ward, and the patient was asked to drink a little water if no postoperative nausea and vomiting (PONV) occurred; otherwise, PONV was treated using nonpharmacological control with or without pharmacological control [16] according to the degrees of discomfort. A small portion of a semifluid diet was encouraged if no PONV occurred. Then, the nutrition procedures could be conducted by specialized nutritionists from the Nutrition Department.

10. Early ambulation evaluation began when the patients could sit up straight 2 hours after returning to the ward. If the patient can keep the straight sitting position without obvious dizziness and weakness, he or she can try to leave the bed and stand beside the sickbed with electrocardiogram monitoring. Marching on the spot can be started if the vital signs and respiratory status remain stable, and then moderate ambulation can be tried with the accompaniment of medical staff and a family member.

11. A single tube was used for the patients in the ERAS group, and the chest tube was removed when the following conditions were met (1): no progressive bleeding or chylothorax (2); no persistent air leakage during continuous cough (3); no obvious atelectasis confirmed by physical examination or imaging examination; and (4) daily serous effusion less than 300 ml/24 h, with the highest volume not exceeding 450 ml/24 h.

12. A multimodal analgesic regimen is recommended for pain relief (16, 17). Opioids are inevitable in most cases, but they should be minimized. Patient education is important but not enough for anxious patients or patients suffering from severe pain, needing psychological counseling and needing additional techniques such as transcutaneous electrical nerve stimulation (TENS).

### Withdrawal from the trial

Patients were withdrawn for one of the following reasons: (i) a withdrawal request was made by the patient or the family member; (ii) there was poor compliance with the training protocol; or (iii) severe complications, such as heart disease or stroke, occurred.

### Sample size calculation

Several primary or secondary outcomes have been studied; however, postoperative pulmonary complications (PPCs) are an important and widely used index for evaluating short-term postoperative improvement (26). The criteria for PPCs were established according to the STS/ESTS definitions (27). It has been reported that the ERAS group has a lower incidence of PPC than the routine group (15.2% vs. 19.5%, P=0.022) (28). For this trial, a minimum 10% absolute risk reduction from a 19.5% PPC risk was set (25). A significant difference between groups was detected with a sample of 480 patients (p = 0.05, 80% power of test, two-sided test), considering a 20% inflation in the case of dropouts and a final sample size of 600.

### Outcome measures and statistical analysis

The primary outcome measures were PPCs. The secondary outcome measures were some other postoperative short-term effects, postoperative long-term respiratory function, health-related quality of life (HRQoL), progression-free survival (PFS) and overall survival (OS) for patients who underwent VATS for lung cancer surgery. Continuous variables are expressed herein as means and standard deviations (SDs); data that were not normally distributed are presented as medians and ranges; and binary variables are presented as proportions. The data were evaluated through Student’s t test (data matching a normal distribution), nonparametric statistics, the chi-square test, or Fisher’s exact test, as appropriate. Multivariate logistic regression analysis was used to reveal the predictors of drainage removal on the first day after surgery without pneumonia during hospitalization. Statistical analysis and graph generation were performed with Stata 12.0 (StataCorp LP, College Station, TX, USA) at a significance level of 0.05. Multivariate logistic regression analysis was performed with SPSS statistics software (version 25; SPSS, Inc., Chicago, IL).

## Results

### Patient characteristics

From July 2020 to June 2022, 611 patients were enrolled in the study; 305 patients were assigned to the ERAS group, and 306 were assigned to the routine group. The treatment protocol was completed for the enrolled patients. The patient characteristics were balanced between the two groups (**Table 1**).

**Table 1:** Patients’ characteristics of the study population.

Characteristics	ERAS group (=305)	Routine group (n=306)	P value
Age, years			0.671
Median	58	59	
Interquartile range	51-66	53-66	
Range	18-83	28-83	
Gender, n (%)			0.654
Male	140 (45.9%)	146 (47.7%)	
Female	165 (54.1%)	160 (52.3%)	
Smoking status, n (%)			0.412
Ever	55 (18.0%)	64 (21.0%)	
Never	250 (82.0%)	242 (79.0%)	
Comorbidity			0.913
Hypertension	79	79	
Diabetes mellitus	30	36	
Heart disease	24	20	
Brain vascular disease	20	22	
COPD^a^	10	9	
Pathological type			0.680
Malignant tumor	264	269	
Adenocarcinoma	200	210	
Squamous cell carcinoma	58	51	
Other	6	8	
Benign tumor	33	32	
Hamartoma	11	4	
Granuloma or inflammation	20	26	
Sclerosing pneumocytoma	2	2	
Pulmonary bulla	8	5	
T stage for Malignant tumor			0.723
Tis	7	9	
T1mi	31	43	
T1	152	143	
T2	51	53	
T3	20	17	
T4	3	4	
N stage for Malignant tumor			0.698
N0	225	222	
N1	25	30	
N2	14	17	
LNs^b^ station dissected			0.201
Median	4	5	
Interquartile range	4-5	4-6	
Range	1-10	2-8	
LNs^b^ amount dissected			0.183
Median	8	9	
Interquartile range	6-13	7-13	
Range	2-32	3-36	
Pulmonary function			
FEV1^c^ (L)			0.210
Median	2.28	2.24	
Interquartile range	1.94-2.73	1.88-2.70	
Range	1.11-4.85	1.05-4.78	
MVV ^d^ (L)			0.485
Median	91.2	89.6	
Interquartile range	79.4-103.2	78.9-103.8	
Range	63.8-118.5	64.6-115.9	
Surgical procedures			0.061
Lobectomy	163	184	
Segmentectomy	55	35	
Wedge resection	87	87	
Operation time (minutes) mean ± SD			0.150
Median	60	60	
Interquartile range	45-90	45-88	
Range	20-240	25-240	
Intraoperative blood loss (mL), mean ± SD			0.946
Median	50	50	
Interquartile range	40-60	40-60	
Range	20-500	20-400	
VATS surgery converted			0.452
to open (%)	9 (3.0%)	6 (2.0%)	

a COPD, chronic obstructive pulmonary disease.

b LNs, lymph nodes.

c FEV1, forced expiratory volume in 1.0 s.

d MVV, maximum volume for ventilation.

As shown in **Table 2**, 210 patients (68.9%) were able to drink water 2 hours after returning to the general ward in the ERAS group, 103 patients (33.8%) had a semifluid diet within 3 hours after returning to the general ward in the ERAS group, and 114 patients (37.4%) had moderate postoperative ambulation on the surgery day with guidance and an accompanying medical staff member and family member. The following three characteristics were significantly different between the ERAS group and the routine group: The duration of drainage in the ERAS group was significantly shorter than that in the routine group (median, 2 days with IQR 1-2 days vs. median, 5 days with IQR 4-6 days). The median postoperative hospital stay was 3 days (IQR, 2-4 days) in the ERAS group, and the median postoperative hospital stay was significantly longer (7 days, IQR, 6-8 days; P <0.001) in the routine group. The readmission rate within 14 days after surgery was similar: 2.0% (6 patients) in the ERAS group and 1.0% (3 patients) in the routine group (P=0.340). No mortality within 30 days occurred in both groups.

**Table 2:** Postoperative recovery of our study population.

Characteristics	ERAS group (n=305)	Routine group (n=306)	P value
Water feeding 2 hours after RTW^a^ (%)	210 (68.9%)	0 (%)	<0.001
Semi- fluid diet within 3 hours after RTW^a^ (%)	103 (33.8%)	0 (%)	<0.001
Postoperative moderate ambulation on the surgery day (%)	114 (37.4%)	7 (2.3%)	<0.001
Duration of drainage (day)			<0.001
Median	2	5	
Interquartile range	1-2	4-6	
Range	1-8	2-11	
Postoperative hospital stay (day)			<0.001
Median	3	7	
Interquartile range	2-4	6-8	
Range	1-13	2-15	
Request extension of hospital stay without objective complications (%)	61 (20.0%)	32 (10.5%)	0.001
Readmission rate within 14 days after surgery (%)	6 (2.0%)	3 (1.0%)	0.340

^a^RTW, Return to ward.

Postoperative blood samples were collected and tested two days after surgery (**Table 3**) unless the patients were discharged from the hospital the first day after surgery (10 patients in the ERAS group). The postoperative white blood cell (WBC) count, percentage of neutrophil granulocytes (NEUT%), and postoperative C-reactive protein (CRP) level were significantly different between the ERAS group and the routine group (p = 0.032; p <0.001; p = 0.023, respectively). Postoperative procalcitonin (PCT) was not significantly different between the two groups (P=0.566). The median albumin (ALB) level was 38.1 in the ERAS group (IQR, 35.4-40.7 g/L) and 37.6 in the routine group (IQR, 34.7-39.5 g/L; P=0.010). The median prealbumin (PA) level was 20.5 in the ERAS group (IQR, 17.6-23.2 mg/dL) and 15.5 in the routine group (IQR, 13.0-18.7 mg/dL; P<0.001).

**Table 3:** Blood laboratory examination of our study population.

Characteristics	ERAS group(n=305)	Routine group (n=306)	P value
Postoperative white blood cell (10^9)			0.032
Median	11.75	12.26	
Interquartile range	10.2-13.83	10.55-14.91	
Range	7.1-25.82	7.01-25.17	
Postoperative NEUT%^a^			<0.001
Median	82.1	84.4	
Interquartile range	79.8-85.5	81.3-86.9	
Range	58.5-94.9	63.3-94.6	
Postoperative CRP^b^ (mg/L)			0.023
Median	109.0	117.1	
Interquartile range	66.2-149.9	86.2-150.0	
Range	18.1-267.7	19.2-263.1	
Postoperative PCT^c^ (ng/ml)			0.566
Median	0.234	0.186	
Interquartile range	0.10-1.427	0.105-0.37	
Range	0.02-10.16	0.02-6.86	
Postoperative ALB^d^ (g/L)			0.012
Median	38.1	37.6	
Interquartile range	35.4-40.68	34.7-39.5	
Range	21.4-51.4	23.9-52.5	
Postoperative PA^e^(mg/dL)			<0.001
Median	20.5	15.5	
Interquartile range	17.6-23.2	13.0-18.7	
Range	5.7-30.4	4.0-27.9	

^a^NEUT%, percentage of neutrophile granulocytes.

^b^CRP, C-reactive protein.

^c^procalcitonin.

^d^albumin

^e^prealbumin

The postoperative early complications are shown in **Table 4**. For pulmonary complications (PPCs), 11.5% of the patients in the ERAS group had pulmonary complications, 22.9% of the patients in the routine group had pulmonary complications, and the difference was significant (P<0.001). There were greater incidences of atelectasis and air leakage in the routine group (P=0.046; P=0.003). Bronchopleural fistula and respiratory failure rarely occurred in either group. The occurrence of supraventricular arrhythmia, acute myocardial infarction, acute cerebral stroke, and venous thromboembolism was similar in both groups. Significantly more urinary irritation was observed in the routine group (ERAS group: 6.2%, routine group: 25.5%; P<0.001), and the frequency of acute urinary retention was similar between the two groups (ERAS group: 0.7%, routine group: 1.6%; P=0.449).

**Table 4:** Postoperative early complications of our study population.

Characteristics	ERAS group (n=305)	Routine group (n=306)	P value
PPCs ^a^ (%)	35 (11.5%)	70 (22.9%)	<0.001
Pneumonia	17 (5.6%)	27 (8.8%)	0.163
Atelectasis	13 (4.3%)	25 (8.2%)	0.046
Air leak (≥5 days)	6 (2.0%)	22 (7.2%)	0.003
Bronchopleural fistula	1 (0.3%)	1 (0.3%)	1.000
Respiratory failure	2 (0.7%)	3 (1.0%)	1.000
Supraventricular arrhythmia (%)	7 (2.3%)	12 (3.9%)	0.348
Acute myocardial infarction (%)	0 (0%)	1 (0.3%)	1.000
Acute cerebral stroke (%)	1 (0.3%)	2 (0.7%)	1.000
VTE^b^ (%)	0 (0%)	2 (0.7%)	0.497
Urinary irritation (%)	19 (6.2%)	78 (25.5%)	<0.001
Acute urinary retention (%)	2 (0.7%)	5 (1.6%)	0.449

^a^PPCs, pulmonary complications. Four patients in ERAS group had ≥ 2 PPCs, eight patient in Routine group had ≥ 2 PPCs.

^b^venous thromboembolism, including deep venous thrombosis (DVT) and pulmonary thromboembolism (PTE).

Multivariate logistic regression analysis was used to reveal the predictors of PPCs. **Table 5** shows that age (P=0.014), ERAS management (P=0.004) were significant predictors. Gender and surgical procedures seemed to have the trend, but neither of them showed the significant statistical difference (P=0.062, P=0.066 respectively).

**Table 5:** Predictors of PPCs.

Predictor ^a^	β value	Odds ratio (95% CI)	P value
Gender	0.634	1.884 (1.024- 3.468)	0.062
Age	0.039	1.040 (1.008- 1.073)	0.014
Smoking status	0.124	1.132 (0.641- 2.000)	0.669
Comorbidity	0.216	1.241 (0.893- 1.724)	0.197
Convert to open	0.052	1.062 (0.317- 3.132)	0.932
Surgical procedures	0.358	1.430 (0.977- 2.096)	0.066
Operation time	0.004	1.004 (0.995- 1.012)	0.397
Intraoperative blood loss	0.001	1.001 (0.996- 1.006)	0.800
ERAS management	-0.880	0.415 (0.229- 0.751)	0.004

^a^Multivariate logistic regression analysis: gender (female: 0, male: 1), age (numerical variable), smoking status (never: 0, ever: 1), comorbidity (hypertension, diabetes mellitus, heart disease, brain vascular disease, COPD, no comorbidity: 0, one comorbidity: 1, two comorbidities: 2, ≥3 comorbidities: 3), Convert to open (no: 0, yes: 1), surgical procedures (wedge resection: 0, segmentectomy: 1, lobectomy: 2), operation time (minutes, numerical variable), intraoperative blood loss (ml, numerical variable), ERAS management (no: 0, yes: 1), PPCs (no PPCs: 0, PPCs:1).

Multivariate logistic regression analysis was also used to reveal the predictors of drainage tube removal on the 1st day after surgery without causing pneumonia during hospitalization. **Table 6** shows that comorbidity (P=0.029), surgical procedure (P=0.001) and operation time (P=0.039) were significant predictors. Rapid removal of chest drainage tubes and recovery without pneumonia may constitute the foundation of day surgery (from admission to discharge ≤48 hours).

**Table 6:** Predictors of removal of the drainage the 1^st^ day (without pneumonia) after surgery.

Predictor ^a^	β value	Odds ratio (95% CI)	P value
Comorbidity			0.029
No comorbidity	Ref.	1	
One comorbidity	0.518	1.678 (0.905-3.112)	
two comorbidities	0.971	2.639 (0.551-12.641)	
≥3 comorbidities	1.204	3.333 (1.346- 8.254)	
Surgical procedures			0.001
Wedge resection	Ref.	1	
Segmentectomy	0.338	1.403 (0.629- 3.126)	
Lobectomy	1.182	3.261 (1.706- 6.232)	
Operation time	0.010	1.010 (1.000- 1.019)	0.039

^a^Multivariate logistic regression analysis: gender (female: 0, male: 1), age (numerical variable), smoking status (never: 0, ever: 1), comorbidity (hypertension, diabetes mellitus, heart disease, brain vascular disease, COPD, no comorbidity: 0, one comorbidity: 1, two comorbidities: 2, ≥3 comorbidities: 3), surgical procedures (wedge resection: 0, segmentectomy: 1, lobectomy: 2), operation time (minutes, numerical variable), intraoperative blood loss (ml, numerical variable), removal of the drainage the 1st day (without pneumonia) (satisfied: 0, not satisfied:1).

## Discussion

In recent years, increasing attention has been given to the implementation of ERAS protocols for various tumors (29–31). The ERAS program for lung surgery (16, 17) was described relatively late and was aimed to decrease postoperative morbidity and mortality.

In the traditional postoperative management mode, patients were asked to stay in the supine posture without drinking or eating for at least 6 hours. Patients were not required to remain in the supine position or fast for a long period when the combination of regional and general anesthetic techniques was used. Patients in the ERAS group were allowed to drink water and have a semifluid diet so that specialized nutritionists could use an earlier intervention. Even more than one-third of the patients in the ERAS group achieved moderate ambulation on the day of surgery, and this approach has been confirmed to be feasible and safe (32). Although contrary to the ERAS protocol, the chest drainage tube could be removed when the volume was less than 100 ml in the traditional mode. However, we suggest careful consideration of early removal of the drainage system, as this approach is relied on successful high-quality minimally invasive surgery, early oral feeding and ambulation. In this study, the ERAS group had a median postoperative hospital stay of 3 days (interquartile range: 2-4 days), which was still shortened gradually.

The biological impact, which was also investigated in our study, has been investigated in several other organ surgeries within the ERAS program (33–35). Blood laboratory examinations, including measurements of biomarker levels indicating the magnitude of surgical stress (36–38), were used in our study. In ERAS group, the postoperative white blood cell count, percentage of neutrophil granulocytes (NEUT%), C-reactive protein (CRP), were significantly lower than those in the routine group, and the levels of albumin (ALB), prealbumin (PA) were significantly higher for patients in the ERAS group. Similar results were obtained in liver surgery (35). Procalcitonin (PCT) increases during severe generalized bacterial, parasitic, or fungal infections (39). Moreover, there was no significant difference in postoperative PCT between the two groups, perhaps due to the similar incidence rate of pneumonia (ERAS group: 5.6%, routine group: 8.8%, P=0.163).

In our study, we found lower incidences of PPCs in the ERAS group than in the control group, and the same results were observed for atelectasis and air leakage (≥5 days). We suppose that early mobilization is crucial for reducing morbidity, promoting lung recruitment as soon as possible, promoting quick recovery of gastrointestinal function, and preventing deep venous thrombosis. Rogers LJ (19) also regarded early mobilization as the most important predictor. ERAS protocols tended to decrease the occurrence of pneumonia, although the difference was not significant. Urinary irritation was significantly lower in the ERAS group (6.2% vs. 25.5%, P<0.001), and acute urinary retention rarely occurred in the ERAS group, even for patients without perioperative transurethral catheters. Of the two patients with acute urinary retention, one man had urinary retention due to prostatitis, and the other patient, a woman, had urinary retention due to unknown reasons; she was cured after urinary catheterization.

We recommend that patients with fewer comorbidities, a smaller resection range, or less operation time be candidates for day surgery (from admission to discharge ≤48 hours), although the management details still need exploration and optimization.

The results of this study were similar to some other studies (19, 28), and for postoperative hospital stay, PPCs like pneumonia and atelectasis, even better results were got in our study. The data couldn’t be used to compare directly due to the difference of enrolled population and implementation details among these studies, but the positive effect of ERAS was confirmed. Furthermore, we propose the concept of multi-gradient individual ERAS (MGI-ERAS). This means that the ERAS protocol is comprehensively formulated and performed in a gradient manner according to the individual conditions of the institutions, anesthetists, surgeons, nurses and patients. This approach may be convenient for medical centers attempting to gradually implement the ERAS protocol. The suggested practice elements of the MGI-ERAS are shown in **Table 7**. The recommendations of Gradient 1 are relatively easier to follow, and the recommendations of Gradient 3 may be the ultimate ERAS practice at present.

**Table 7:** Practice elements recommendations of multi- gradient individual ERAS (MGI- ERAS).

Items	Gradient 1	Gradient 2	Gradient 3
Preadmission information, education and counsellin	Simply completed during simple guidan c	ERAS group makes the simple guidan c	Specialized ERAS group makes systematic guidance on nutrition, smoking cessation, alcohol dependency management, and pulmonary prehabilitation
Airway management after admission	Climbing stairs and simple physical pulmonary training guided by surgeons or nurses	ERAS group guided the preoperative airway management	ERAS group including a specialized physiotherapist guided the preoperative airway management
Aspirin withdrawal or not (routinr minimally invasive surgery with low bleeding ris)	Aspirin withdrawal for at least one we	Aspirin withdrawal for 3-5 days then LMWH^a^ bringing management	No aspirin withdrawal
Preoperative fasting and carbohtdrate treatment	Clear fluids be allowed up until 4 hours before the induction of anaesthesia and soilids until 8 hours before induction	Oral carbohydrate loading be allowed up until 2-4 hours before the induction of anaesthesia and solids until 8 hours before induction	Oral carbohtdrate loading be allowed up until 2 hoursv before the induction of anaesthesia and solids until 6 hours before induction
Preventing intraoperative Hypothermi	Temperature control of the operating room during surgery	Convective active warming devices used perioperatively	Continuous measurement of core temperature to guide the temperature control of the patients.
Urinary drainage (routine minimally invasive segmentectomy surgery without need for strict fluid management)	The transurethral catheter is moved immediately after the operation	Not routinely placed for patients undergo wedge resection	Not routinely placed for lobectomy, even for sleeve lobectomy or tracheal surgery with low bleeding ris
Anaesthetic protoco	A combination of regional and general anaesthetic techniques with lung protective strategies during one-lung ventilation	Completely tubeless protocol^b^ for patients undergo wedge resection.	Completely tubeless protocol^b^ for segmentectomy, lobectomy, even for sleeve lobectomy or tracheal surgery with low bleeding risk.
Surgical techniqu	Three- port minimally invasive surgery (including VATS and robotic surgery	Two- port minimally invasive surgery (including VATS and robotic surgery)	Uniport minimally invasive surgery (including VATS and robotic surgery)
Postoperative recovery Raising the head of bed (by ≥30°) Sit up straight 4- 6 hours Sit up straight 2 hours after returning to position and mobilization	Raising the head of bed (by ≥30°) immediately back to the general ward, patients should be mobilized within 24 hours of surgery	Sit up straight 4- 6 hours after returning to the general ward, try to leave the sickbed 6- 8 hours after returning to the general ward	Sit up straight 2 hours after returning tothe general ward, moderate ambulationis tried 4 hours after returning to the general ward
Postoperative water drinking and diet	Try water drinking 4- 6 hours after returning to the general ward if no PONV^c^ occurs, semi- fluid diet is permitted	Try water drinking 2- 4 hours after returning to the general ward, if no PONV^c^ occurs, semi- fluid diet is permitted. The nutrition procedures is better conducted by specialized nutritionists	Try water drinking ≤2 hours after returning to the general ward, if no PONV^c^ occurs, semi- fluid diet is permitted. The nutrition procedure is better conducted by specialized nutritionists.
Removal of the chest Tubes	Thoracic closed drainage with negative pressure can be use for continuous air leak, chest tubes should be removed with the daily serous effusion without progressive bleeding or chylothorax)≤200ml.	Thoracic closed drainage with negative pressure can be used for continuous air leak, chest tubes should be removed with the daily serous effusion (without progressive bleeding or chylothorax)≤300ml.	Thoracic closed drainage with negative pressure can be used for continuous air leak, chest tubes should be removed with the daily serous effusion (without progressive bleeding or chylothorax) ≤450ml.
ain relief regim	Multimodal analgesic regime is recommended	Multimodal analgesic regime with the minimized opioids education and pain management consultation is needed sometimes	Multimodal analgesic regime with the minimized opioids dosage, professional psychological counselling for anxious patients and additional techniques such as transcutaneous electrical nerve stimulation carried out by physiotherapist is used.

^a^LMWH, low molecular weight heparin.

^b^Completely tubeless protocol: VATS surgery with both non-intubated intravenous anesthesia and no urinary catheterization protocol.

^c^PONV, postoperative nausea and vomiting.

There are several limitations that should not be ignored. First, this was a single-center study. Second, the patients were enrolled more than 2 years previously, and gains in ERAS experience are inevitable, which may have led to bias. Third, a blinded method could not be used in this study, and the timing of return to diet, mobilization, removal of the chest tube, and discharge from the hospital might have been biased or even affected by the patients or their relatives. Moreover, pain control, psychological variables, postoperative functional recovery efficacy and quality of life were not presented. Finally, the economic outcomes of the ERAS program were not explored in this study.

## Conclusion

In conclusion, the implementation of the ERAS protocol led to earlier return to diet and mobilization, lower incidences of PPCs and urinary irritation without acute urinary retention, and shorter durations of drainage and postoperative hospital stay, thus providing better biological impacts for patients undergoing VATS lung resection. MGI-ERAS is recommended for the implementation of the ERAS protocol at different institutions with respect to the individual conditions of the patients.

## Data Availability

The original contributions presented in the study are included in the article/supplementary material. Further inquiries can be directed to the corresponding authors.
